# Vertical root distribution underpins belowground resistance to extreme drought

**DOI:** 10.1093/nsr/nwag592

**Published:** 2026-09-15

**Authors:** Chong Xu, Yunlong Zhang, Honghui Wu, Qiang Yu, Hongqiang Wang, Yuguang Ke, Wei Zhou, Yingjie Yan, Jiale Chen, Yadong Yang, Tian Yang, Xiaoan Zuo, Wentao Luo, Melinda D Smith, Elizabeth T Borer, Iain P Hartley, Ingrid J Slette, Wenping Yuan, Deliang Kong, Xiaoping Xin, Shikui Dong, Baoming Ji, Liebao Han, Wenju Zhang, Minggang Xu, Guirui Yu, Xingguo Han, Yann Hautier

**Affiliations:** State Key Laboratory of Efficient Utilization of Arable Land in China, Institute of Agricultural Resources and Regional Planning, Chinese Academy of Agricultural Sciences, Beijing 100081, China; Key Laboratory of Arable Land Quality Monitoring and Evaluation, Ministry of Agriculture and Rural Affairs, Institute of Agricultural Resources and Regional Planning, Chinese Academy of Agricultural Sciences, Beijing 100081, China; Ecology and Biodiversity Group, Department of Biology, Utrecht University, 3584 CH Utrecht, The Netherlands; College of Grassland Science and Technology, China Agricultural University, Beijing 100193, China; State Key Laboratory of Efficient Utilization of Arable Land in China, Institute of Agricultural Resources and Regional Planning, Chinese Academy of Agricultural Sciences, Beijing 100081, China; Key Laboratory of Arable Land Quality Monitoring and Evaluation, Ministry of Agriculture and Rural Affairs, Institute of Agricultural Resources and Regional Planning, Chinese Academy of Agricultural Sciences, Beijing 100081, China; School of Grassland Science, Beijing Forestry University, Beijing 100083, China; State Key Laboratory of Efficient Utilization of Arable Land in China, Institute of Agricultural Resources and Regional Planning, Chinese Academy of Agricultural Sciences, Beijing 100081, China; Key Laboratory of Arable Land Quality Monitoring and Evaluation, Ministry of Agriculture and Rural Affairs, Institute of Agricultural Resources and Regional Planning, Chinese Academy of Agricultural Sciences, Beijing 100081, China; State Key Laboratory of Efficient Utilization of Arable Land in China, Institute of Agricultural Resources and Regional Planning, Chinese Academy of Agricultural Sciences, Beijing 100081, China; Key Laboratory of Arable Land Quality Monitoring and Evaluation, Ministry of Agriculture and Rural Affairs, Institute of Agricultural Resources and Regional Planning, Chinese Academy of Agricultural Sciences, Beijing 100081, China; State Key Laboratory of Forage Breeding-by-Design and Utilization, Key Laboratory of Vegetation and Environmental Change, Institute of Botany, Chinese Academy of Sciences, Beijing 100093, China; School of Grassland Science, Beijing Forestry University, Beijing 100083, China; School of Grassland Science, Beijing Forestry University, Beijing 100083, China; State Key Laboratory of Efficient Utilization of Arable Land in China, Institute of Agricultural Resources and Regional Planning, Chinese Academy of Agricultural Sciences, Beijing 100081, China; Key Laboratory of Arable Land Quality Monitoring and Evaluation, Ministry of Agriculture and Rural Affairs, Institute of Agricultural Resources and Regional Planning, Chinese Academy of Agricultural Sciences, Beijing 100081, China; Department of Biology, The University of Western Ontario, London, ON N6A 5B7, Canada; State Key Laboratory of Ecological Safety and Sustainable Development in Arid Lands, Urat Desert-grassland Research Station, Northwest Institute of Eco-Environment and Resources, Chinese Academy of Sciences, Lanzhou 730000, China; Erguna Forest-Steppe Ecotone Research Station, Institute of Applied Ecology, Chinese Academy of Sciences, Shenyang 110164, China; Department of Biology, Colorado State University, Fort Collins, CO 80523, USA; Department of Ecology, Evolution, and Behavior, University of Minnesota, St. Paul, MN 55108, USA; Department of Geography, Faculty of Environment, Science and Economy, University of Exeter, Exeter EX4 4RJ, UK; Department of Biology, Colorado State University, Fort Collins, CO 80523, USA; Institute of Carbon Neutrality, College of Urban and Environmental Sciences, Peking University, Beijing 100871, China; College of Forestry, Henan Agricultural University, Zhengzhou 450046, China; State Key Laboratory of Efficient Utilization of Arable Land in China, Institute of Agricultural Resources and Regional Planning, Chinese Academy of Agricultural Sciences, Beijing 100081, China; National Hulunber Grassland Ecosystem Observation and Research Station, Institute of Agricultural Resources and Regional Planning, Chinese Academy of Agricultural Sciences, Beijing 100871, China; School of Grassland Science, Beijing Forestry University, Beijing 100083, China; School of Grassland Science, Beijing Forestry University, Beijing 100083, China; School of Grassland Science, Beijing Forestry University, Beijing 100083, China; State Key Laboratory of Efficient Utilization of Arable Land in China, Institute of Agricultural Resources and Regional Planning, Chinese Academy of Agricultural Sciences, Beijing 100081, China; Key Laboratory of Arable Land Quality Monitoring and Evaluation, Ministry of Agriculture and Rural Affairs, Institute of Agricultural Resources and Regional Planning, Chinese Academy of Agricultural Sciences, Beijing 100081, China; Institute of Eco-Environment and Industrial Technology, Shanxi Agricultural University, Taiyuan 030031, China; Key Laboratory of Ecosystem Network Observation and Modeling, Institute of Geographic Sciences and Natural Resources Research, Chinese Academy of Sciences, Beijing 100101, China; College of Resources and Environment, University of Chinese Academy of Sciences, Beijing 101408, China; School of Life Sciences, Hebei University, Baoding 071002, China; Ecology and Biodiversity Group, Department of Biology, Utrecht University, 3584 CH Utrecht, The Netherlands

**Keywords:** extreme climate, grasslands, roots, coordinated distributed experiment

## Abstract

Extreme droughts are becoming more frequent and strongly affect ecosystem productivity, yet their impact on belowground productivity—key to the carbon cycle—remains underexplored. Using a coordinated drought experiment across six Eurasian grassland sites spanning a precipitation gradient, we found that extreme drought had little effect on belowground net primary productivity (BNPP) or root biomass across the 0–20 cm soil profile because drought-induced shifts in root allocation compensated across soil depths. Specifically, drought induced site- and depth-specific shifts: in xeric grasslands, BNPP and root biomass increased in the 0–10 cm layer but declined in the 10–20 cm layer, whereas mesic grasslands exhibited the opposite pattern. These contrasting responses were consistent with shifts in depth-specific soil moisture. Consequently, the apparent resistance of belowground productivity to drought masks a redistribution of root growth along the vertical soil profile. Accounting for such depth-dependent root dynamics is essential for understanding ecosystem functioning, including carbon cycling, plant persistence and ecosystem responses to intensifying climate extremes.

## INTRODUCTION

The frequency and intensity of climate extremes are predicted to increase with climate change [1]. Despite their relatively short duration, climate extremes can induce significant and long-lasting ecological changes, thereby exerting impacts that are disproportionate to their temporal scale [2–4]. Among all climate extremes, drought is anticipated to have the most acute and lasting effects on grassland ecosystems, owing to the critical role of precipitation as a principal limiting resource [5–7]. For example, the carbon cycle in grassland ecosystems is strongly influenced by drought, which disrupts key processes such as plant productivity, thereby altering ecosystem functioning [8–10]. Moreover, growing-season drought can have particularly strong impacts on grasslands [11]. Therefore, many studies have focused on how substantial reductions in growing-season precipitation limitation affect grassland ecosystems [7,12]. However, previous studies have largely focused on aboveground productivity, overlooking the response of belowground productivity, even though most primary productivity occurs belowground and accounts for a larger carbon flux in grassland ecosystems [13,14]. As models

forecast a rising frequency of extreme droughts [1,15,16], it is imperative to gain a deeper understanding of belowground productivity responses. Such understanding is essential for advancing carbon cycle models, thereby improving our capacity to predict the effects of extreme drought on carbon dynamics.

Grassland ecosystems exhibit considerable variation in their responses to extreme drought, largely due to inherent differences in their biotic and abiotic characteristics [7,9,12]. One compelling explanation involves variation in mean annual precipitation (MAP), leading to two contrasting hypotheses: the resource limitation hypothesis, which posits that xeric sites respond more sensitively due to limited resources and the stress adaptation hypothesis, which suggests that mesic sites are more sensitive because their plant communities are less drought-adapted [7]. Previous studies on aboveground productivity generally support the resource limitation hypothesis [9,17]. A growing body of experimental studies and syntheses has also examined how drought affects belowground productivity, showing that belowground responses are often context-dependent across ecosystems [18,19]. Recent syntheses have further demonstrated that root responses to precipitation changes differ between shallow and deep soil layers, with shallow root biomass generally declining, while responses in deeper layers are maintained or even enhanced [20,21]. However, it remains unclear whether and how these depth-specific responses vary among grassland ecosystems across broad precipitation gradients, and whether such ecosystem-specific patterns persist through time under extreme drought. This distinction is important because roots at different soil depths access different water and nutrient pools, while the vertical distribution of belowground productivity varies across grassland types and precipitation regimes [22,23]. Drought-induced shifts in this vertical distribution may influence plant persistence and productivity maintenance by altering access to soil resources [24,25], redistribute root-derived carbon inputs among soil layers and affect soil carbon cycling [26] and ecosystem responses to increasingly intense drought. Thus, understanding how depth-specific responses to drought vary among grassland ecosystems is crucial for interpreting divergent belowground productivity responses and their ecological consequences.

Soil moisture provides a potential pathway linking drought to depth-specific root responses. Because roots can respond to spatial variation in soil water availability, depth-specific changes in soil moisture under drought may be associated with corresponding changes in root biomass and productivity [27–29]. In xeric ecosystems, where moisture recharge in deeper layers is limited during extreme drought, plants may respond by concentrating root growth near the surface and reducing deeper root productivity to cope with the lack of water at depth [30,31]. In contrast, mesic ecosystems, characterized by higher moisture availability in deeper soils, may experience the opposite pattern: decreased shallow productivity and increased allocation of roots to deeper layers [32]. However, these responses may also be reversed or more complex due to the diverse changes of soil moisture [33]. Therefore, variation in background climatic water availability and depth-specific soil moisture responses may help explain differences in drought-induced vertical root redistribution among ecosystems. While these contrasting strategies are plausible, they have yet to be tested systematically using standardized experimental treatments across contrasting grassland ecosystems.

Although soil moisture may be closely associated with depth-specific root responses to drought, additional biotic and abiotic factors may also contribute to variation in these responses across ecosystems. Dominant species and plant diversity can influence rooting strategies and resource acquisition, while nutrient availability and soil texture may alter water availability and root allocation patterns [34–37]. These factors may therefore contribute to ecosystem-specific variation in belowground drought responses, but their relative importance remains unclear.

Here, we investigate total and depth-specific belowground net primary productivity (BNPP at 0–10 and 10–20 cm; root biomass at 0–10, 10–20 and 20–40 cm) in response to experimentally imposed extreme drought, defined here as a sustained 66% reduction in growing-season precipitation, and evaluate how these responses are associated with biotic (dominant species cover, species richness) and abiotic factors (MAP, soil moisture, inorganic nitrogen and soil texture) across six grasslands in Inner Mongolia, China. These sites represent major grassland types in the eastern Eurasian region, including xeric desert grasslands (Urat, URA; Sheila MuRen, MUR), typical grasslands (Xilinhot-*Stipa grandis*, XLS; Xilinhot-*Leymus chinensis*, XLL) and mesic meadow grasslands (Erguna, ERG; Sher Tara, TAR), and span a broad precipitation gradient (see Methods and Table S1). Our Extreme Drought in Grasslands Experiment (EDGE) used a randomized block design with six replicate blocks at each site and paired ambient and drought plots within each block [12]. The drought treatment was imposed from 2015 to 2018 by reducing each precipitation event during the growing season by 66%, enabling robust cross-ecosystem comparisons of global change impacts [38,39]. BNPP was measured from 2016 to 2018 using ingrowth cores [40], whereas root biomass was sampled annually from 2015 to 2018 using soil cores. By combining a standardized 4-year extreme-drought treatment with repeated measurements across six contrasting grasslands, our study extends existing evidence for depth-specific root responses by assessing whether vertical redistribution differs among grassland ecosystems, which soil-moisture and plant-community factors are associated with these responses, and whether the observed patterns remain consistent across years. These questions are difficult to address through single-site manipulative experiments, which cannot directly compare responses among ecosystems, or through global syntheses, in which differences in experimental design, treatment intensity, sampling depth, measurement methods and study duration may limit direct comparisons of ecosystem-specific and temporal responses [38,39]. We hypothesized that apparent resistance of total root biomass and BNPP to drought would emerge through compensatory redistribution across soil depths, with drought increasing shallow-layer root biomass or BNPP but reducing deeper-layer values in xeric grasslands, while mesic grasslands would exhibit the reverse pattern, with these patterns hypothesized to arise in association with depth-specific changes in soil moisture content (Fig. 1).

**Figure 1.**
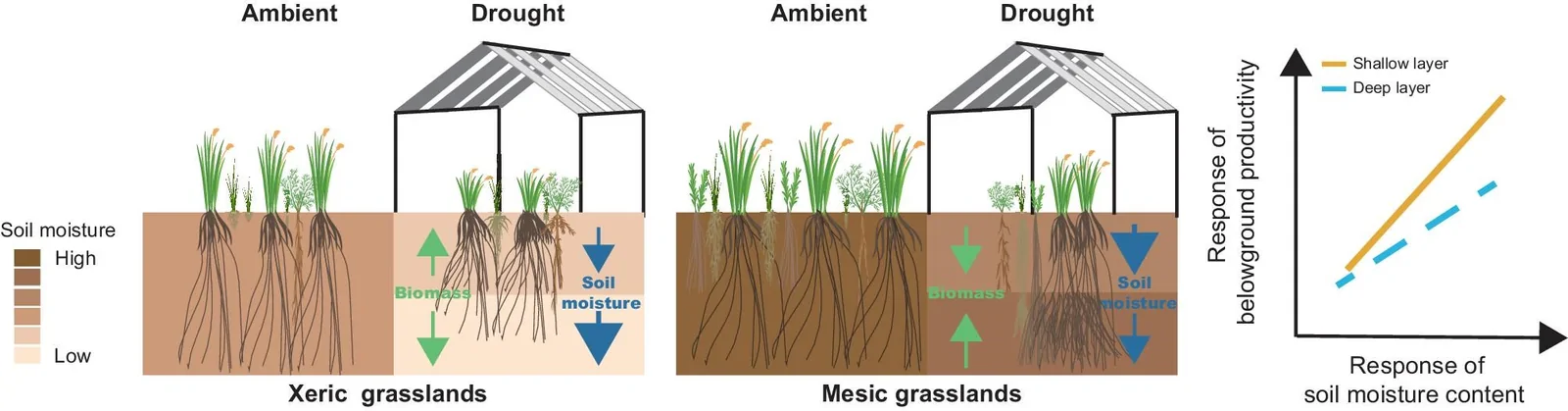
Conceptual diagram illustrating drought-induced changes in belowground productivity across dry (xeric) vs wetter (mesic) grassland types and soil depths. The arrows for belowground productivity and soil moisture represent their responses to extreme drought, respectively. Upward and downward arrows indicate positive and negative effects. Larger arrows indicate stronger effects.

## RESULTS

### Belowground productivity response to extreme drought

Over the 4-year experimental period, extreme drought had little effect on total BNPP (except at XLS) or root biomass (except at XLL) in the 0–20 cm layer (Fig. 2; Figs S1 and S2; Tables S2 and S3). However, drought effects on the vertical distribution of BNPP and root biomass within the 0–20 cm soil profile were strongly site-dependent. Root biomass in the 20–40 cm (except at ERG) and 0–40 cm (except at XLL) layers remained generally unaffected (Fig. 2; Figs S1 and S2; Table S3). Although the magnitude of drought responses varied among years, year-specific results generally supported the multi-year average patterns of depth-dependent redistribution (Fig. 2; Fig. S1; Tables S2 and S3).

**Figure 2.**
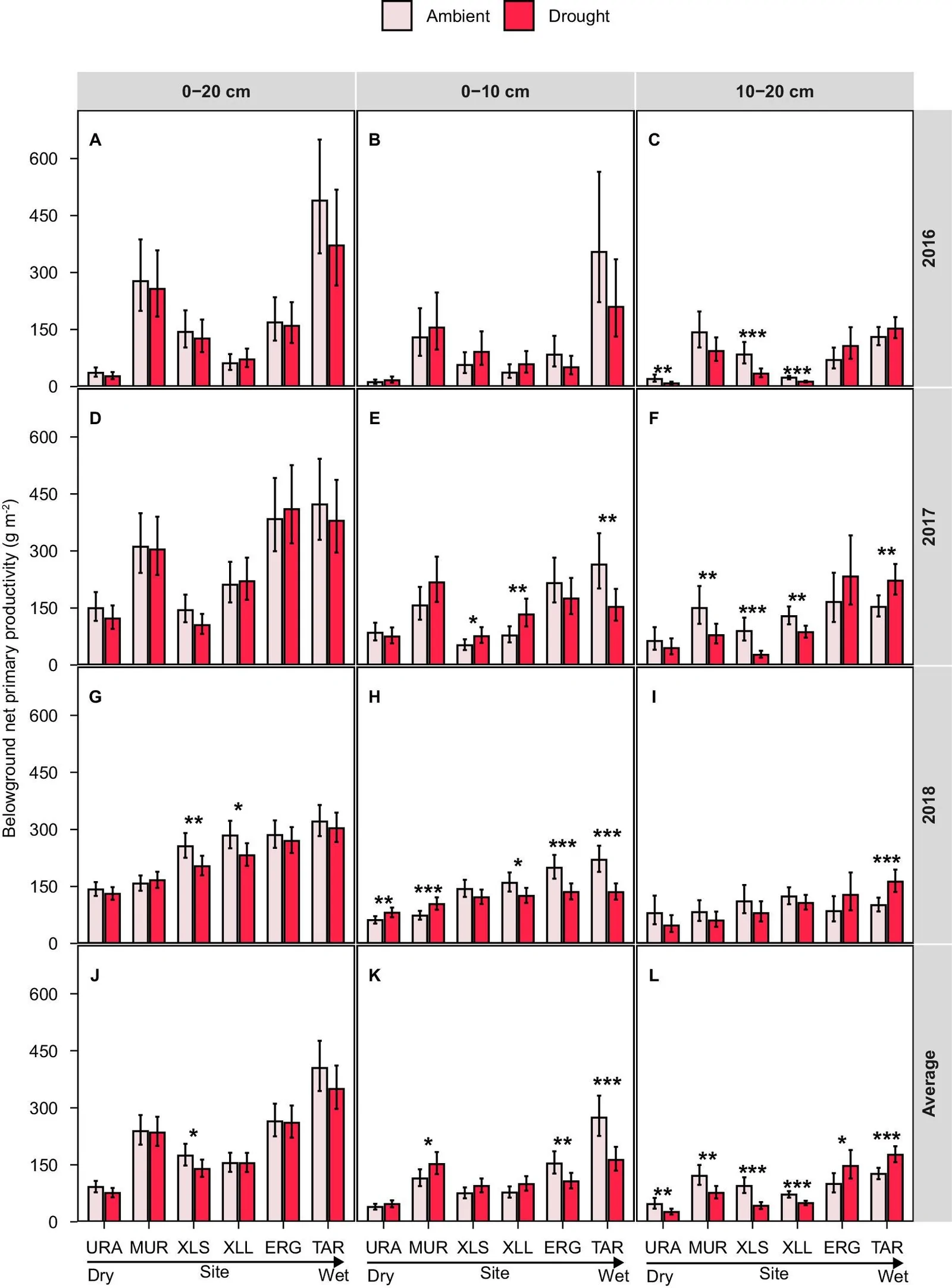
Effects of extreme drought on BNPP across soil depth, years and sites. Panels show BNPP in the 0–20, 0–10 and 10–20 cm soil layers in 2016 (A–C), 2017 (D–F), 2018 (G–I) and the multi-year average (J–L). Results were estimated using linear mixed-effects models. Values represent mean ± standard error (SE). Asterisks indicate significant differences between ambient and drought treatments within each site. Significance levels are denoted as ***, *P* < 0.001; **, *P* < 0.01; and *, *P* < 0.05.

In the 0–10 cm soil layer, BNPP generally increased in the drier grasslands, including a significant increase at MUR (+30.06%) and non-significant increases at URA (+15.98%), XLS (+15.77%) and XLL (+15.64%), whereas significant declines occurred at the wetter sites ERG (−30.65%) and TAR (−37.69%) (Fig. 2K). A similar pattern was observed for root biomass in the 0–10 cm layer, with significant increases at MUR (+43.17%), XLS (+41.23%) and XLL (+60.40%), a non-significant increase at URA (+14.19%), and significant decreases at ERG (−32.26%) and TAR (−28.87%) (Fig. S1U).

In contrast, BNPP and root biomass in the 10–20 cm layer showed the opposite trend to 0–10 cm layer. BNPP significantly declined at MUR (−36.28%), XLS (−50.60%), XLL (−26.14%) and URA (−28.21%), whereas significant increases at ERG (+43.11%) and TAR (+41.16%) (Fig. 2L). Root biomass in this layer followed a similar pattern, with significant declines at MUR (−33.97%), XLS (−23.95%) and XLL (−28.14%), a non-significant decrease at URA (−3.47%), whereas it showed non-significant increases at ERG (+40.46%) and TAR (+23.87%) (Fig. S1V).

### Linkages between biotic and abiotic factors and belowground productivity responses

Extreme drought reduced soil moisture content in both the 0–10 and 10–20 cm layers, but the magnitude of the reduction varied among sites and soil depths (Fig. 3A; Fig. S3M and N). In the 0–10 cm layer, the decline in soil moisture content increased along the precipitation gradient, from the driest to the wettest sites (Fig. 3A; Fig. S3M). In contrast, in the 10–20 cm layer, reductions in soil moisture content became more pronounced from the wettest to the driest sites (Fig. 3A; Fig. S3N). Within-site drought response ratios of soil moisture content were positively associated with the corresponding response ratios of BNPP and root biomass in both the 0–10 and 10–20 cm soil layers (Fig. 3B; Table S4). Soil moisture content in the 20–40 cm layer did not differ significantly between ambient and extreme drought treatments (Fig. S3O).

**Figure 3.**
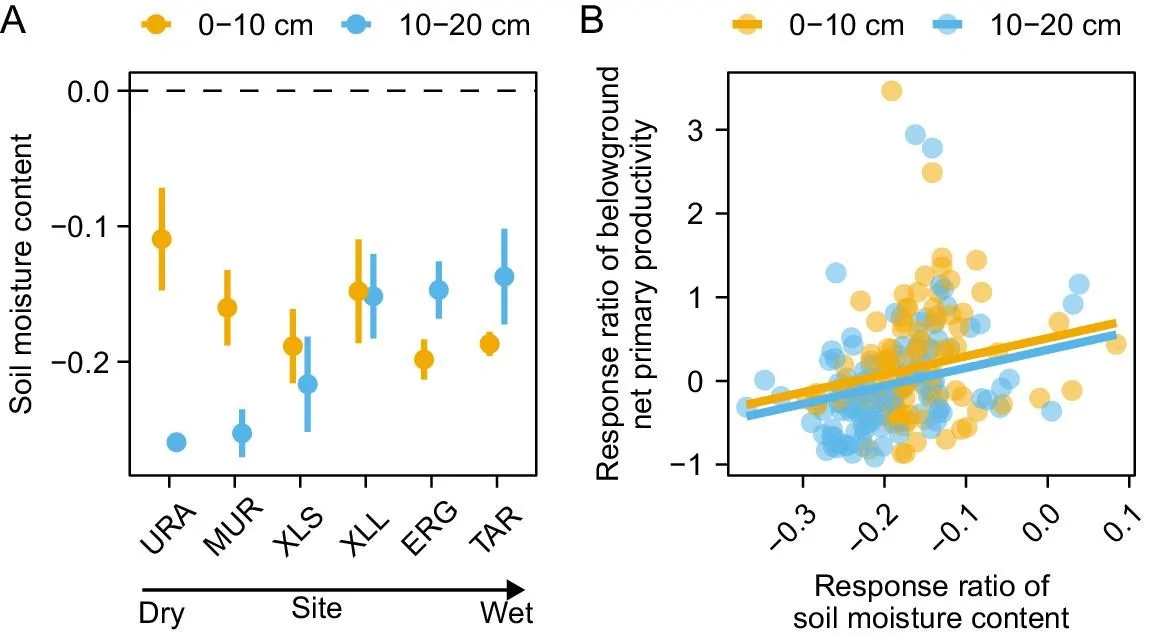
Relationships between soil moisture responses and belowground productivity responses to extreme drought across soil depths. Panel A shows response ratios of soil moisture content in the 0–10 and 10–20 cm soil layers across the six study sites; values represent mean ± 95% confidence intervals, and separate series represent the two soil depths. Panel B shows relationships between soil moisture response ratios and BNPP response ratios across soil depths. Each point represents an individual plot-year observation, with points shown separately for the two soil depths. Solid lines represent fitted relationships from linear mixed-effects models. Models accounted for the hierarchical experimental design and repeated measurements by including site and block as random effects and temporal autocorrelation among years where appropriate. See Table S2 for detailed model specifications and summary statistics.

Extreme drought had no significant effect on soil texture (clay, silt and sand content) across all depths (Fig. S3A–I), and generally did not affect soil inorganic nitrogen content, except for increases in the 0–10 cm layer at ERG and TAR and in the 10–20 cm layer at XLS and XLL (Fig. S3J–L). Dominant species cover declined under drought at all sites (Figs S3P and S4A), while species richness was unaffected except at URA and ERG (Figs S3Q and S4B). No significant relationships were found between the response ratios of dominant species cover or species richness and those of root biomass or BNPP in either the 0–10 or 10–20 cm layer (Fig. S5B, C, E and F; Table S4).

Piecewise structural equation models (SEMs) showed that the conditional associations among drought treatment, soil moisture content, dominant species cover, species richness and BNPP or root biomass varied between soil layers and response variables (Fig. 4; Fig. S6). Soil moisture content showed the most consistent association with BNPP and root biomass across models. Drought generally reduced soil moisture content, and lower soil moisture content was associated with lower belowground productivity, while associations involving dominant species cover and species richness were weaker and less consistent. Year effects were retained in the final models but did not alter the overall pattern that drought effects on belowground productivity were more consistently associated with soil moisture content than with plant-community attributes.

**Figure 4.**
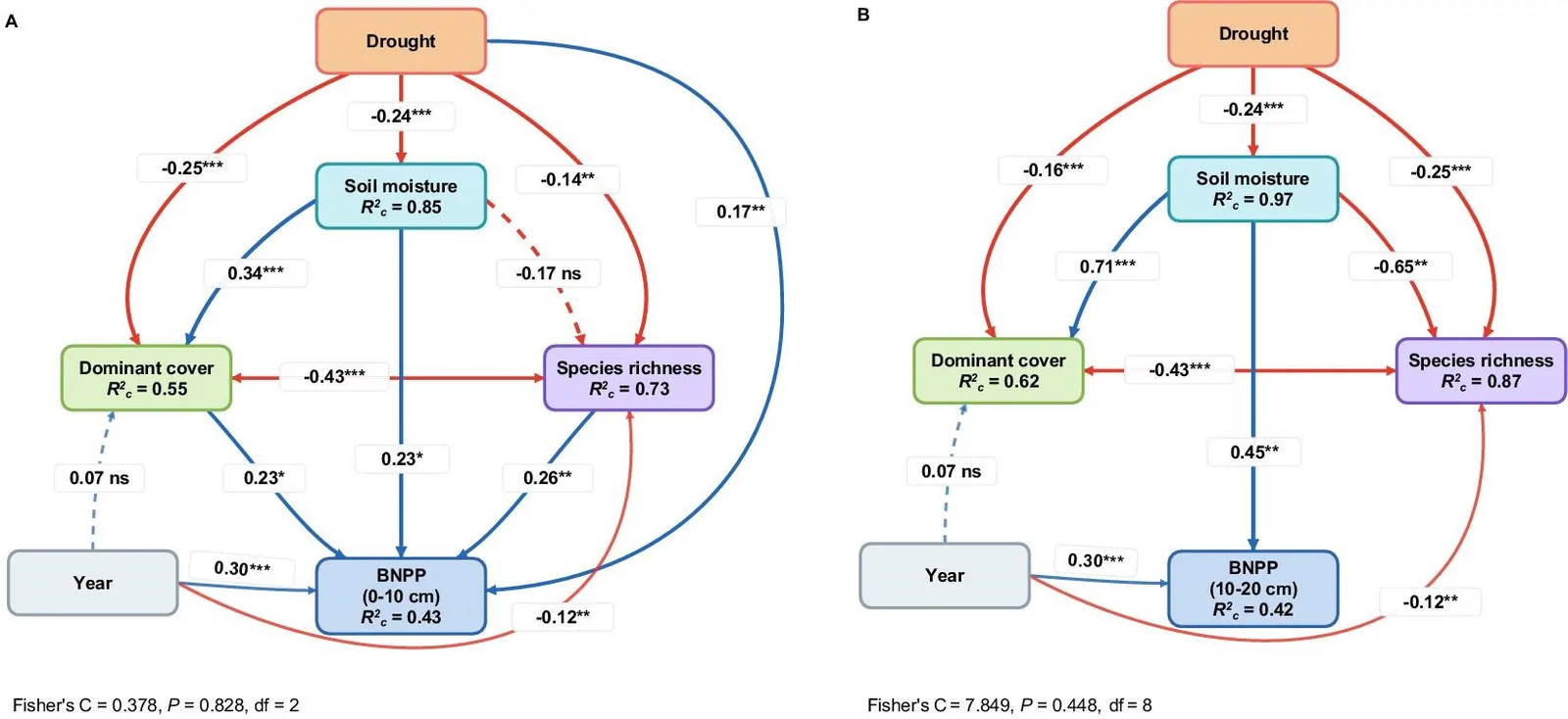
Piecewise structural equation models describing the conditional associations among extreme drought treatment, soil moisture content, dominant species cover, species richness and BNPP across years. Models are shown for BNPP in the 0–10 cm soil layer (A) and 10–20 cm soil layer (B). Arrows indicate paths retained in the final models. The sign of each standardized path coefficient indicates whether the association is positive or negative. Solid arrows indicate significant paths (*p* < 0.05). Values beside arrows are standardized path coefficients. Model fit statistics based on Fisher’s *C* test are shown in each panel. Significance levels are denoted as ***, *P* < 0.001; **, *P* < 0.01; and *, *P* < 0.05.

MAP was negatively associated with the within-site drought response ratios in the 0–10 cm layer but positively associated with response ratios in deeper layers, including the 10–20 cm layer for both BNPP and root biomass and the 20–40 cm layer for root biomass (Fig. S5A and D; Table S5).

## DISCUSSION

We examined the effects of extreme drought on total and depth-specific belowground productivity, together with the potential biotic and abiotic factors associated with these responses, across six representative Eurasian grassland sites under a standardized 4-year drought treatment—addressing a key knowledge gap in whether depth-specific belowground responses to extreme drought are consistent across contrasting grassland ecosystems. We found that apparent belowground resistance to extreme drought emerged through compensatory redistribution of roots across soil depths, with the direction of redistribution depending on site climate. In drier grasslands, drought increased root biomass and BNPP in the shallow layer but reduced the biomass of the deeper root layer, whereas roots in wetter grasslands exhibited the opposite pattern. These opposing depth-specific responses were largely compensatory, such that apparent resistance of total BNPP and root biomass emerged through redistribution rather than invariance in belowground productivity across the precipitation gradient. These ecosystem-specific patterns were generally maintained across years, although their magnitudes varied. Across sites and soil depths, changes in soil moisture were most consistently associated with the responses of BNPP and root biomass to drought. These findings and their ecological implications are discussed in detail in the following sections.

### Stable totals but shifting root distribution under extreme drought

Extreme drought had little effect on either total BNPP (0–20 cm) or root biomass (0–20 and 0–40 cm) at nearly all sites (Fig. 2; Figs S1 and S2; Tables S2 and S3), indicating that belowground productivity in these grasslands remains stable under such conditions. This finding contrasts with previous studies, which have generally reported strongly negative impacts of extreme drought on grassland primary productivity, particularly in drier regions [7,41]. One major reason for this discrepancy is that most prior research has focused predominantly on aboveground productivity, while the responses of belowground components have been largely overlooked [9,12]. From a belowground perspective, our results provide compelling evidence that grassland ecosystems exhibit strong apparent resistance to extreme drought that was associated with shifts in vertical root distribution. These insights complement and extend our current understanding of drought impacts on grassland functioning by providing new insights into root depth redistribution in differing climates and soils.

Extreme drought reshaped root vertical distribution in ecosystem-specific ways. In drier grasslands, it increased shallow (0–10 cm) BNPP and root biomass but decreased them at 10–20 cm, whereas wetter grasslands showed the opposite pattern (Fig. 2; Figs S1 and S2; Tables S2 and S3). Within the measured soil profile, drought effects were strongest in the upper 20 cm, whereas root biomass at 20–40 cm showed very limited response to drought (Fig. S1; Table S3). This pattern suggests that vertical redistribution was concentrated in the upper soil layers measured in this study. These contrasting responses explain the high overall resistance of belowground productivity to drought and reveal divergent root allocation strategies: dry grasslands favor shallow rooting, while those that are more mesic shift roots deeper. While prior site-level studies noted such patterns [30,42], our standardized, multi-site experiment provides stronger evidence for the generality of ecosystem-specific strategies in vertical root allocation under extreme drought. Importantly, although drought responses varied in magnitude among years, the overall direction of depth-specific redistribution was broadly maintained, indicating that the observed vertical reorganization was not simply a transient year-specific response. Our findings suggest that apparent belowground resistance to drought should not be interpreted as an absence of response, but rather as evidence of compensatory reorganization of root allocation across the soil profile.

### Soil moisture content is closely linked to belowground productivity responses

Drought-induced reductions in soil moisture content varied with depth and site climate, with greater declines in the 0–10 cm layer at mesic sites and in the 10–20 cm layer at xeric sites (Fig. 3A; Fig. S3M and N). Furthermore, the within-site drought response ratios of root biomass and BNPP were positively associated with the corresponding response ratios of soil moisture content in both the 0–10 and 10–20 cm layers (Fig. 3B; Table S4). These findings are consistent with previous studies suggesting that soil moisture content plays an important role in shaping belowground productivity responses to extreme drought [27–29]. This interpretation was further supported by the SEMs, in which soil moisture showed the strongest and most consistent standardized associations with BNPP and root biomass, whereas dominant species cover and species richness showed weaker and less consistent conditional associations (Fig. 4; Fig. S6). Although soil moisture and belowground productivity were measured during the same periods and the SEMs may reflect both within-site treatment variation and among-site environmental differences, the agreement between the SEMs and the within-site response-ratio analyses strengthens the inference that soil moisture was the measured factor most consistently associated with depth-specific belowground productivity responses. This convergence is important because the two complementary analytical approaches are based on different sources of variation, with the SEMs integrating variation among sites and treatments and the response-ratio analyses focusing on paired drought responses within sites and blocks. In contrast, the within-site drought response ratios of dominant species cover and species richness were not significantly associated with the corresponding response ratios of BNPP or root biomass (Fig. S3P and Q; Fig. S5B, C, E and F). Previous studies suggest that drought-induced changes in soil water availability can simultaneously influence plant communities and root growth, thereby affecting root allocation, competition for water and belowground productivity [27,30,42]. Together, these complementary results support soil moisture as an important potential pathway linking drought to depth-specific belowground productivity responses.

Variations in environmental factors across ecosystems—particularly those related to soil moisture—regulate root distribution across different soil depths [22]. In xeric grasslands, limited precipitation, infrequent rainfall events and restricted infiltration can constrain water recharge of deeper soil layers, making drought effects on soil moisture more pronounced in the 10–20 cm layer [30,31]. In contrast, in mesic grasslands, greater background water availability can maintain relatively wetter deeper soils, whereas the surface layer becomes more vulnerable to drought-induced drying, resulting in larger reductions in soil moisture in the 0–10 cm layer [32]. These contrasting depth-specific soil moisture responses may help explain the ecosystem-specific patterns of belowground productivity observed in this study. Consistent with this interpretation, drought-induced reductions in soil moisture content were greatest in the 10–20 cm layer at xeric sites but in the 0–10 cm layer at mesic sites, matching the depth at which the strongest shifts in BNPP and root biomass occurred (Figs 2 and 3; Figs S1 and S2). This depth-dependent pattern provides a potential framework for reconciling previously inconsistent findings on belowground drought responses across environmental contexts. Rather than a uniform drought response, our results suggest that belowground responses vary with drought-induced changes in soil moisture availability across soil depths and climatic settings. Additionally, extreme drought did not alter soil moisture content in the 20–40 cm layer (Fig. S3O), which may further help explain why root biomass in this layer remained largely unchanged (Fig. S1).

We also examined whether broader climatic and soil background variables were associated with belowground productivity response to extreme drought, including MAP [7], inorganic nitrogen [36] and soil texture [37]. The relationship between MAP and belowground productivity responses was strongly depth-dependent. In the shallow layer, the response ratios of both BNPP and root biomass were negatively correlated with MAP, whereas in deeper layers the relationships became positive (Fig. S5A and D; Table S5). In contrast to most studies that report a positive relationship between aboveground net primary productivity (ANPP) responses to drought and MAP [5,41,43], our results indicate that the association between MAP and belowground productivity responses vary with soil depth. Wetter sites tended to show stronger reductions in shallow belowground productivity but weaker reductions, or even increases, in deeper soil layers. However, inorganic nitrogen and soil texture showed weak or inconsistent responses to drought and did not match the depth-specific patterns observed for BNPP and root biomass (Fig. S3A–L). Therefore, the response of belowground productivity to extreme drought was most consistently associated with variation in soil moisture content across sites and soil depths, further supporting an important potential role for soil moisture in explaining variation in root allocation responses under different drought regimes and climatic conditions [28].

### Implications and future directions

Our findings provide critical insights for advancing carbon cycle models of grassland ecosystems and improving the predictive capacity for carbon dynamics under extreme climate conditions. We highlight the need for current ecosystem models—designed to simulate carbon uptake, allocation and plant growth—to incorporate the dynamic responses of roots to drought to more accurately represent terrestrial carbon cycling [44,45]. Most terrestrial ecosystem models still assume a static asymptotic fine-root distribution [46], characterized by an exponential decline in root density with depth and a fixed vertical distribution of BNPP irrespective of soil moisture variability [47]. In contrast, our findings show that the vertical distributions of root biomass and BNPP were strongly associated with spatial and temporal variation in soil moisture. Neglecting these vertical adjustments may cause models to overestimate the detrimental impacts of drought on vegetation growth. Furthermore, uncertainties in simulating root distributions impair the ability to accurately reproduce vertical patterns of root litterfall, substantially affecting estimates of soil respiration [48]. We therefore emphasize the urgent need to incorporate root distributions and their covariation with soil moisture into terrestrial ecosystem models to more accurately capture plant adaptive strategies under environmental stress and to better predict ecosystem carbon fluxes under future climate extremes [49].

Several limitations should be considered to guide future investigations. First, our inference about whole-profile belowground responses is constrained by sampling depth. Root biomass was measured to 40 cm, whereas BNPP was measured only to 20 cm using ingrowth cores. Although these depths capture the major rooting zone in many grasslands [33,47,50], and root biomass at 20–40 cm showed no significant drought response in our study, root productivity > 20 cm may still respond to drought [30]. Therefore, our BNPP results should be interpreted as upper-soil responses rather than whole-profile BNPP responses. Future studies with deeper BNPP measurements are needed to evaluate potential deeper-root responses under drought. Second, the geographic scope, six Eurasian grasslands, may limit broader inference. While our results align with other Eurasian and temperate sites [30,32], they contrast with some North American grasslands, where drought reduces belowground productivity and promotes deeper deep rooting in drier areas [51], suggesting the possibility for regional or biome-specific responses. Distributed experiments across regions and continents provide an opportunity to test this. Third, the 4-year drought duration constrains understanding of long-term effects. As extreme droughts become more frequent [1,15,16], legacy effects may emerge [52,53], potentially reducing the resistance and capacity for vertical redistribution that we observed. Fourth, we did not fully assess shifts in plant community composition or species-level root biomass. Because community composition is a key driver of root traits, water use and competition [28,52,53], incorporating species-level root measurements would help identify the relative roles of species turnover, interspecific variation in drought sensitivity and within-species allocation shifts in shaping depth-specific belowground responses. Identifying species-specific strategies and incorporating community dynamics will be critical for advancing mechanistic understanding of belowground drought responses.

## CONCLUSIONS

Our results demonstrate that total BNPP and root biomass appeared resistant to extreme drought because drought-induced shifts in vertical root distribution compensated across soil depths. This work further reveals that the direction of vertical root redistribution differed between xeric and mesic grasslands and was associated with depth-specific soil moisture responses. These ecosystem-specific patterns were generally maintained across years, although their magnitudes varied. Together, these findings show that apparent belowground resistance to drought reflects compensatory redistribution rather than invariance in productivity, highlighting the importance of considering ecosystem-specific patterns of depth-dependent root responses together with soil moisture dynamics. Such vertical shifts have ecological implications beyond belowground productivity itself, as they may influence plant persistence, soil carbon cycling and ecosystem responses to increasingly intense drought.

## METHODS

### Experimental designs

The EDGE in China were conducted in six grasslands [12]. These encompassed the major types of Eurasian grasslands with desert grasslands located in URA and Sheila MUR, typical grasslands located in XLS and XLL, as well as meadow grassland located in ERG and Sher TAR. These six sites span a broad precipitation gradient and differ in dominant species, species richness and soil texture (Table S1).

The EDGE used a randomized block design. At each site, identical manipulative drought experiments were established in relatively homogeneous areas in 2014. All six sites included ambient plots (ambient precipitation) and plots with extreme drought—imposed as a 66% reduction of each precipitation event throughout the growing season (from May to August) from 2015 to 2018. Rainout shelters equipped with strips of corrugated polycarbonate were used to passively reduce precipitation events [54]. The drought treatment was classified as extreme (<5th percentile of historical precipitation amounts) based on a probability density function of precipitation calculated from 37 years (1982–2018) of MAP data [12]. Each site contained six replicate blocks, with two 6 m × 6 m plots per block, each located at least 2 m from neighboring plots. Each plot included a 1 m external buffer to allow access to the plot and to minimize the edge effect associated with the infrastructure.

### Root biomass and BNPP

Root biomass was collected at the end of the growing season each year of the drought (2015–2018). Because temperate grasslands generally have relatively shallow rooting profiles, with most roots concentrated in the upper soil layers (<30 cm) [33,47,50], we focused root biomass sampling on the upper 40 cm and BNPP measurements on the upper 20 cm to characterize depth-specific responses in the primary rooting zone. In each experimental plot, two soil cores (7 cm in diameter) were extracted to a depth of 40 cm and divided into three depth increments: 0–10, 10–20 and 20–40 cm. Roots were washed with clean water over a 0.5 mm mesh sieve to remove adhering soil. Root biomass was initially measured to a depth of 40 cm during the first year of the experiment; however, responses in the 20–40 cm soil layer were much weaker than those observed in the upper 20 cm. Therefore, from the second year onward, BNPP measurements were focused on the 0–20 cm soil layer. Root biomass, however, continued to be sampled to a depth of 40 cm throughout the experiment to maintain consistency with the original sampling design and to provide complementary information on root distribution across soil depths. BNPP was measured in 2016, 2017 and 2018 using the ingrowth core method [40]. At the beginning of each growing season, two soil cores (5 cm in diameter and 20 cm in depth) were collected from each plot. Each core was divided into two soil layers (0–10 and 10–20 cm), and all visible roots were manually removed from the collected soil. The root-free soil from each depth was then placed into separate nylon mesh bags (mesh size < 1 mm) and reinserted into the original holes in the same plot. At the end of the growing season, the nylon mesh bags were retrieved intact, and the soil columns within each bag were separated into 0–10 and 10–20 cm layers. Newly produced roots were separated from the soil and washed with clean water over a 0.5-mm mesh sieve. Root samples were oven-dried at 65°C for 48 h and weighed. BNPP at each soil depth was quantified as the dry mass of roots produced within the ingrowth cores during the growing season.

### Biotic and abiotic factors

Plant cover and species richness were measured at the end of the growing season each year of the drought (2015–2018). A randomly designated 2 × 2 m area divided into four 1 × 1 m subplots within each rainout shelter and ambient plot was permanently marked for the measurement of plant cover and species richness. The cover of individual plant species rooted in each permanently marked subplot was estimated visually to the nearest 1% annually at the same time as the measurement of species richness. We defined dominant species as those with a relative frequency >0.8 (80%) and a relative cover greater than 12%, and the rest as subordinate species [12]. The number of species in each subplot was recorded annually at peak biomass and these subplots were averaged to determine species richness in each plot.

Soil moisture content in the 0–10 and 10–20 cm soil layers was measured at the end of the growing season each year of the drought (2015–2018). Soil moisture content in the 20–40 cm soil layer, inorganic nitrogen and soil texture was measured at the end of the growing season in 2024. Soil moisture content in the 0–10 and 10–20 cm soil layers was measured with soil moisture time-domain reflectometer probes (CS616, Campbell Scientific, Logan, UT, USA) at 30-min intervals, inserted diagonally (45°) in the center of each plot. We collected soil samples from each plot to determine soil moisture content, inorganic nitrogen, and clay, silt and sand contents. Soil moisture content in the 20–40 cm soil layer was determined from the mass ratio of the water to solids by drying the samples in an oven at 105°C for 48 h. Inorganic nitrogen (ammonium nitrogen and nitrate nitrogen) in the 0–10, 10–20 and 20–40 cm soil layers was extracted from soil samples using 2 M KCl and quantified using a flow injection analyzer and total inorganic nitrogen was calculated as the sum of ammonium nitrogen and nitrate nitrogen. Clay, silt and sand contents in the 0–10, 10–20 and 20–40 cm soil layers were measured on sieved (<2 mm), air-dried soil samples using laser diffraction with a Beckman Coulter LS 13 320 analyzer (Beckman Coulter, Indianapolis, USA).

Climate data for each studied site was retrieved from China Meteorology Administration (http://data.cma.cn/) and the National Climate Data Center of the National Oceanic and Atmospheric Administration (NOAA) (http://www.ncdc.noaa.gov/). Long-term precipitation data from 1982 to 2018 were used to calculate MAP for each site. The aridity index (MAP/mean annual potential evapotranspiration) [55] for each site was calculated using estimates from World Clim v2 [56].

### Statistical analysis

To examine drought effects on BNPP and root biomass, we fitted mixed-effects analysis of variance (ANOVA) models separately for each response variable. We first analyzed total BNPP or root biomass using models that included treatment, site, year and their interactions as fixed effects, with site-block included as a random effect. Year was treated as a categorical variable. The model structure was response variable ~ treatment × site × year + (1|site-block). We then fitted models with the same fixed- and random-effects structure separately for each soil layer to examine depth-specific responses. *Post hoc* comparisons between ambient and drought treatments were conducted within each site and year using Tukey-adjusted pairwise comparisons. BNPP and root biomass were log-transformed to improve the normality of model residuals. Model assumptions were evaluated using residual diagnostics, including residuals-versus-fitted plots and Q–Q plots of residuals. When residual diagnostics indicated heteroscedasticity, we incorporated variance structures using the weights argument in the *lme* function. To account for repeated measurements through time, we also evaluated temporal autocorrelation by comparing models with and without a first-order autoregressive correlation structure, AR(1). The AR(1) structure was specified for repeated observations within each site-block-treatment combination. Final variance and correlation structures were selected based on Akaike information criterion corrected for small sample size (AICc) comparisons and likelihood-ratio tests. The selected structures for each model are reported in Table S7. We also fitted models for total root biomass at 0–20 cm to make comparisons with the results from total BNPP (0–20 cm). Because analyses of BNPP and root biomass showed similar patterns, we report BNPP results in the main text and provide root biomass results in the supplementary materials.

To further investigate the mechanisms underlying drought effects, we tested whether drought altered key abiotic and biotic factors. These variables included soil moisture content measured at 0–10 and 10–20 cm across 4 years (2015–2018) and at 20–40 cm in 2024; dominant species cover and species richness measured across 4 years (2015–2018); and inorganic nitrogen, clay, silt and sand contents measured at 0–10, 10–20 and 20–40 cm in 2024. For each response variable, we fitted mixed-effects models including treatment, site, year where available, soil layer where available, and their interactions as fixed effects, with site-block included as a random effect. As above, we evaluated alternative variance structures where appropriate, and selected final model structures based on AICc comparisons and likelihood-ratio tests (see Table S8 for detailed model structures). We further calculated response ratios ((extreme drought – ambient)/ambient) for BNPP at 0–10 and 10–20 cm, root biomass at 0–10, 10–20 and 20–40 cm, soil moisture content at 0–10 and 10–20 cm, dominant species cover and species richness. We then tested whether the response ratios of BNPP and root biomass were associated with the response ratios of soil moisture content, dominant species cover and species richness, and whether these relationships differed between soil layers. For this analysis, we fitted mixed-effects models that included the predictor response ratios, soil layer and their interactions as fixed effects, with block nested in site included as a random effect, and temporal autocorrelation among years was accounted for where appropriate.

To explore the associations among drought treatment, soil moisture, plant community structure and BNPP or root biomass, we constructed a series of piecewise SEMs using the package [57]. Because soil moisture and belowground productivity were measured during the same periods, their causal direction could not be determined. Moreover, because the models combined observations from multiple sites, the estimated associations may reflect both within-site drought treatment variation and among-site differences in climate, soil properties, vegetation composition and climatic history. We therefore interpreted the path coefficients as conditional associations rather than evidence of direct causal relationships. Separate models were developed for the 0–10 and 10–20 cm soil layers based on our *a priori* expectation that drought treatment, soil moisture, plant community structure and belowground productivity would be interrelated (Fig. S7). The SEM included drought treatment, soil moisture content, dominant species cover, species richness and BNPP/root biomass. Year was included as a continuous fixed effect in the component models, and site-block was included as a random effect to account for the experimental design. Model fit was evaluated using Fisher’s *C* statistic, with a non-significant Fisher’s *C* test indicating adequate model fit. Standardized path coefficients were used to compare the relative strengths of the conditional associations represented in the fitted models.

To evaluate whether broader climatic context was associated with belowground productivity responses to extreme drought, we analyzed relationships between the response ratios of belowground productivity and MAP. MAP was treated as a site-level climatic background variable. These relationships were tested using mixed-effects models with MAP, soil layer and their interaction as fixed effects, and block nested within site as a random effect: response ratio ~ MAP × soil layer + (1|site/block). All statistical analyses and figures were conducted in R v.4.3.1.

## Supplementary Material

nwag592_Supplemental_File
