# Supplementary material for: Vertical root distribution underpins belowground resistance to extreme drought
Source: Natl Sci Rev. 2026 Sep 15;13(20):nwag592. doi: 10.1093/nsr/nwag592 (PMC13767667; doi:10.1093/nsr/nwag592)
Supplement: Supplementary file 1 [file nwag592_supplemental_file.docx]

**Supporting Information for**

**Vertical root distribution underpins belowground resistance to extreme drought**

**This PDF file includes**

Supplementary Tables 1-8

Supplementary Figures 1-7

**Table S1.** Study site and climate information.

| Site name | URA | MUR | XLS | XLL | ERG | TAR |
| --- | --- | --- | --- | --- | --- | --- |
| Latitude | 41°25'N | 41°47'N | 43°32'N | 43°33'N | 50°10'N | 49°21'N |
| Longitude | 106°58'E | 111°53'E | 116°33'E | 116°40'E | 119°22'E | 120°06'E |
| Aridity index | 0.1 | 0.15 | 0.25 | 0.26 | 0.38 | 0.38 |
| MAT (℃) | 6 | 4.4 | 1.1 | 1.1 | 0 | -0.5 |
| MAP (mm) | 174 | 248 | 333 | 333 | 366 | 395 |
| Grassland type | Desert | Desert | Typical | Typical | Meadow | Meadow |
| Dominant species | *Stipa glareosa* | *Leymus chinensis, S. breviflora* | *S. grandis, L. chinensis, Cleistogenes squarrosa* | *L. chinensis, S. grandis, Achnatherum sibiricum* | *L. chinensis, Carex duriuscula* | *L. chinensis, C. pediformis* |
| Species richness (number m ^-2^) | 4 | 11 | 7 | 8 | 12 | 10 |
| Clay content (%) | 6.27 | - | 5.42 | 4.42 | 4.71 | 4.80 |
| Silt content (%) | 21.78 | - | 27.76 | 25.41 | 23.82 | 25.25 |
| Sand content (%) | 71.95 | - | 66.82 | 70.18 | 71.47 | 69.95 |
| Inorganic nitrogen content (mg kg ^-1^) | 20.55 | - | 27.09 | 29.96 | 19.04 | 23.03 |

URA, MUR, XLS, XLL, ERG and TAR represent the sites Urat, Sheila MuRen, Xlingol-*Stipa grandis*, Xlingol-*Leymus chinensis*, Erguna and Sher Tara, respectively. MAT (℃) and MAP (mm) represent mean annual temperature and mean annual precipitation, respectively. The MAT and MAP were calculated based on 37 years of data (1982-2018). The aridity index was calculated as mean annual precipitation / mean annual potential evapotranspiration (see Methods).

**Table S2.** Summary of linear mixed-effects models assessing relationships testing drought effects on BNPP. Block for each site was included as a random effect, and temporal autocorrelation among years was accounted for where appropriate. Results are reported using Type II sums of squares.

| Response variable | Predictor | *Chisq* | *Df* | *P*-value |
| --- | --- | --- | --- | --- |
| BNPP  (0-20 cm) | Site | 345.03 | 5 | **< 0.001** |
|  | Treatment | 11.86 | 1 | **< 0.001** |
|  | Year | 118.56 | 2 | **< 0.001** |
|  | Site: Treatment | 8.61 | 5 | 0.13 |
|  | Site: Year | 321.62 | 10 | **< 0.001** |
|  | Treatment: Year | 0.03 | 2 | 0.99 |
|  | Site: Treatment: Year | 6.46 | 10 | 0.78 |
| BNPP  (0-10 cm) | Site | 274.80 | 5 | **< 0.001** |
|  | Treatment | 2.79 | 1 | 0.10 |
|  | Year | 67.33 | 2 | **< 0.001** |
|  | Site: Treatment | 68.10 | 5 | **< 0.001** |
|  | Site: Year | 234.88 | 10 | **< 0.001** |
|  | Treatment: Year | 5.16 | 2 | 0.08 |
|  | Site: Treatment: Year | 25.19 | 10 | **< 0.01** |
| BNPP  (10-20 cm) | Site | 382.38 | 5 | **< 0.001** |
|  | Treatment | 8.86 | 1 | **< 0.01** |
|  | Year | 343.03 | 2 | **< 0.001** |
|  | Site: Treatment | 78.34 | 5 | **< 0.001** |
|  | Site: Year | 457.18 | 10 | **< 0.001** |
|  | Treatment: Year | 12.61 | 2 | **< 0.01** |
|  | Site: Treatment: Year | 10.57 | 10 | 0.39 |

**Table S3.** Summary of linear mixed-effects models assessing relationships testing drought effects on root biomass. Block for each site was included as a random effect, and temporal autocorrelation among years was accounted for where appropriate. Results are reported using Type II sums of squares.

| Response variable | Predictor | *Chisq* | *Df* | *P*-value |
| --- | --- | --- | --- | --- |
| Root biomass  (0-40 cm) | Site | 554.79 | 5 | **< 0.001** |
|  | Treatment | 1.14 | 1 | 0.29 |
|  | Year | 138.38 | 3 | **< 0.001** |
|  | Site: Treatment | 6.34 | 5 | 0.28 |
|  | Site: Year | 239.71 | 15 | **< 0.001** |
|  | Treatment: Year | 19.19 | 3 | **< 0.001** |
|  | Site: Treatment: Year | 48.03 | 15 | **< 0.001** |
| Root biomass  (0-10 cm) | Site | 255.91 | 5 | **< 0.001** |
|  | Treatment | 4.48 | 1 | 0.03 |
|  | Year | 27.39 | 3 | **< 0.001** |
|  | Site: Treatment | 59.53 | 5 | **< 0.001** |
|  | Site: Year | 136.76 | 15 | **< 0.001** |
|  | Treatment: Year | 4.86 | 3 | 0.18 |
|  | Site: Treatment: Year | 45.22 | 15 | **< 0.001** |
| Root biomass  (10-20 cm) | Site | 969.06 | 5 | **< 0.001** |
|  | Treatment | 7.06 | 1 | **< 0.01** |
|  | Year | 206.15 | 3 | **< 0.001** |
|  | Site: Treatment | 67.25 | 5 | **< 0.001** |
|  | Site: Year | 368.28 | 15 | **< 0.001** |
|  | Treatment: Year | 19.18 | 3 | **< 0.001** |
|  | Site: Treatment: Year | 28.71 | 15 | **< 0.05** |
| Root biomass  (20-40 cm) | Site | 463.02 | 5 | **< 0.001** |
|  | Treatment | 0.47 | 1 | 0.49 |
|  | Year | 228.46 | 3 | **< 0.001** |
|  | Site: Treatment | 8.11 | 5 | 0.15 |
|  | Site: Year | 290.64 | 15 | **< 0.001** |
|  | Treatment: Year | 3.20 | 3 | 0.36 |
|  | Site: Treatment: Year | 27.69 | 15 | **0.02** |
| Root biomass  (0-20 cm) | Site | 454.75 | 5 | **< 0.001** |
|  | Treatment | 1.36 | 1 | 0.24 |
|  | Year | 78.16 | 3 | **< 0.001** |
|  | Site: Treatment | 8.43 | 5 | 0.13 |
|  | Site: Year | 167.24 | 15 | **< 0.001** |
|  | Treatment: Year | 16.27 | 3 | **< 0.001** |
|  | Site: Treatment: Year | 48.37 | 15 | **< 0.001** |

**Table S4.** Summary of linear mixed-effects models assessing relationships between belowground productivity response ratios and abiotic or biotic response ratios across soil layers and six sites. Block nested within site was included as a random effect, and temporal autocorrelation among years was accounted for where appropriate. Results are reported using Type II sums of squares.

| Response variable | Predictor | *Chisq* | *Df* | *P*-value |
| --- | --- | --- | --- | --- |
| Belowground productivity (BNPP) | Soil moisture content | 11.15 | 1 | **< 0.01** |
|  | Dominant species cover | 0.72 | 1 | 0.40 |
|  | Species richness | 0.03 | 1 | 0.86 |
|  | Layer | 1.45 | 1 | 0.23 |
|  | Soil moisture content: Layer | 0.20 | 1 | 0.65 |
|  | Dominant species cover: Layer | 0.35 | 1 | 0.55 |
|  | Species richness: Layer | < 0.01 | 1 | 0.95 |
| Root biomass (0-10, 10-20 cm) | Soil moisture content | 9.86 | 1 | **< 0.01** |
|  | Dominant species cover | 3.44 | 1 | 0.06 |
|  | Species richness | 0.03 | 1 | 0.87 |
|  | Layer | 4.25 | 1 | **0.04** |
|  | Soil moisture content: Layer | 0.01 | 1 | 0.95 |
|  | Dominant species cover: Layer | 0.86 | 1 | 0.35 |
|  | Species richness: Layer | 0.11 | 1 | 0.74 |

**Table S5.** Summary of linear mixed-effects models assessing relationships between belowground productivity response ratios and mean annual precipitation across soil layers and six sites. Results are reported using Type II sums of squares.

| Response variable | Predictor | *Chisq* | *Df* | *P*-value |
| --- | --- | --- | --- | --- |
| Belowground productivity (BNPP) | Mean annual precipitation | 0.85 | 1 | 0.36 |
|  | Layer | 6.79 | 1 | **< 0.01** |
|  | Mean annual precipitation: Layer | 15.86 | 1 | **< 0.001** |
| Root biomass | Mean annual precipitation | 0.14 | 1 | 0.25 |
|  | Layer | 11.34 | 2 | **< 0.01** |
|  | Mean annual precipitation: Layer | 8.99 | 2 | **0.01** |

**Table S6.** Summary of linear mixed-effects models assessing relationships between belowground productivity and annual precipitation across soil layers and six sites. Results are reported using Type II sums of squares.

| Response variable | Predictor | *Chisq* | *Df* | *P*-value |
| --- | --- | --- | --- | --- |
| Belowground productivity (BNPP) | Annual precipitation | 0.16 | 1 | 0.69 |
|  | Treatment | 1.90 | 1 | 0.17 |
|  | Layer | 26.23 | 1 | **< 0.001** |
|  | Annual precipitation: Treatment | 3.65 | 1 | 0.06 |
|  | Annual precipitation: Layer | 7.20 | 1 | **< 0.01** |
|  | Treatment: Layer | 2.47 | 1 | 0.12 |
|  | Annual precipitation: Treatment: Layer | 0.28 | 1 | 0.60 |
| Root biomass | Annual precipitation | 1.08 | 1 | 0.30 |
|  | Treatment | 1.53 | 1 | 0.22 |
|  | Layer | 798.92 | 2 | **< 0.001** |
|  | Annual precipitation: Treatment | 0.33 | 1 | 0.57 |
|  | Annual precipitation: Layer | 68.24 | 2 | **< 0.001** |
|  | Treatment: Layer | 37.56 | 2 | **< 0.001** |
|  | Annual precipitation: Treatment: Layer | 7.88 | 2 | **0.02** |

**Table S7.** Summary of variance and correlation structures used in mixed-effects models testing drought effects on BNPP and root biomass.

| Response Variable | Predictor | Variance structure | Correlation structure |
| --- | --- | --- | --- |
| Total BNPP | Site × Treatment × Year | varIdent (form = ~1 \| Year) | No |
| BNPP 0-10 cm | Site × Treatment × Year | varIdent (form = ~1 \| Year) | No |
| BNPP 10-20 cm | Site × Treatment × Year | varIdent (form = ~1 \| Site) | Yes |
| Total root biomass | Site × Treatment × Year | varIdent (form = ~1 \| Year) | Yes |
| Root biomass 0-10 cm | Site × Treatment × Year | varIdent (form = ~1 \| Year) | Yes |
| Root biomass 10-20 cm | Site × Treatment × Year | varIdent (form = ~1 \| Year) | No |
| Root biomass 20-40 cm | Site × Treatment × Year | varIdent (form = ~1 \| Year) | No |
| Root biomass 0-20 cm | Site × Treatment × Year | varIdent (form = ~1 \| Year) | Yes |

**Table S8.** Summary of model structures used in mixed-effects models testing drought effects on abiotic and biotic factors.

| Response Variable | Predictor | Variance structure |
| --- | --- | --- |
| Soil clay content | Site × Treatment × Layer | NULL |
| Soil silt content | Site × Treatment × Layer | NULL |
| Soil sand content | Site × Treatment × Layer | NULL |
| Inorganic nitrogen content | Site × Treatment × Layer | NULL |
| Soil moisture content (0-10 and 10-20 cm) | Site × Treatment × Layer × Year | varIdent(form = ~1 \| Year) |
| Soil moisture content (20-40 cm) | Site × Treatment | NULL |
| Dominant species cover | Site × Treatment × Year | varIdent(form = ~1 \| Site) |
| Species richness | Site × Treatment × Year | varIdent(form = ~1 \| Site) |

**Figure S1.** Effects of extreme drought on root biomass across soil depth, years and sites. Root biomass under ambient and drought treatments is shown for the 0-10 cm, 10-20 cm, 20-40 cm, 0-20 cm, and 0-40 cm soil layers in 2015 (A-E), 2016 (F-J), 2017 (K-O), 2018 (P-T), and averaged across treatment years (U-Y). Values represent mean ± SE. Asterisks indicate significant differences between ambient and drought treatments within each site. Significance levels are denoted as ***, *P* < 0.001; **, *P* < 0.01; and *, *P* < 0.05.

**Figure S2.** The response ratios of (A) belowground net primary productivity (BNPP) and (B) root biomass to extreme drought. Error bars show mean ± 95% confidence intervals. Error bars that do not overlap with zero indicate statistically significant effects of extreme drought (*P* < 0.05). Non-overlapping error bars between different sites suggest significant differences in their responses to extreme drought.

**Figure S3.** The effect of extreme drought on biotic and abiotic factors. The response of soil clay content in the 0-10 cm (A), 10-20 cm (B), and 20-40 cm soil layers (C); soil silt content in the 0-10 cm (D), 10-20 cm (E), and 20-40 cm soil layers (F); soil sand content in the 0-10 cm (G), 10-20 cm (H), and 20-40 cm soil layers (I); soil inorganic nitrogen (IN) in the 0-10 cm (J), 10-20 cm (K), and 20-40 cm soil layers (L); soil moisture content in the 0-10 cm (M), 10-20 cm (N) and 20-40 cm soil layer (O); dominant species cover (P); and species richness (Q) to extreme drought. Values represent *mean* ± *SE*. Asterisks indicate significant differences between ambient and drought treatments within each site. Significance levels are denoted as ***, *P* < 0.001; **, *P* < 0.01; and *, *P* < 0.05.

**Figure S4.** Response (expressed as response ratios) of (A) dominant plant species cover and (B) plant species richness to extreme drought. Error bars show mean ± 95% confidence intervals. Error bars that do not overlap with zero indicate statistically significant effects of extreme drought (*P* < 0.05). Non-overlapping error bars between different sites suggest potential differences in their responses to extreme drought.

**Figure S5.** Relationships between the response ratio of belowground net primary productivity (BNPP) and root biomass with mean annual precipitation and the response ratio of biotic factors. The relationship between mean annual precipitation (MAP, A), the response ratios of dominant species cover (B) and species richness (C), and the response ratio of BNPP; and the relationship between MAP (D), the response ratios of dominant species cover (E) and species richness (F), and the response ratio of root biomass. See Table S2 and S3 for detailed model statistics.

**Figure S6.** Piecewise structural equation models describing the conditional associations among extreme drought treatment, soil moisture content, dominant species cover, species richness, and root biomass in the 0–10 cm and 10–20 cm soil layers. Models are shown for root biomass in the 0–10 cm soil layer (A) and 10–20 cm soil layer (B). Arrows indicate paths retained in the final models. Blue and red arrows represent positive and negative associations, respectively. Solid arrows indicate significant paths (P < 0.05). Values beside arrows are standardized path coefficients. Model fit statistics are shown in each panel based on *Fisher’s* C test. Significance levels are indicated as: ***, *P* < 0.001; **, *P* < 0.01; *, *P* < 0.05.

**Figure S7.** A priori structural equation model describing the hypothesized associations among extreme drought treatment, soil moisture content, plant community attributes, and belowground productivity. The model was applied separately to the 0–10 cm and 10–20 cm soil layers for BNPP and root biomass. Arrows indicate the hypothesized directional associations evaluated in the piecewise structural equation models, including associations of extreme drought treatment with belowground productivity, soil moisture content, dominant species cover, and species richness, as well as associations of these biotic and abiotic factors with belowground productivity.
